# Expanding the genotypic spectrum of combined oxidative phosphorylation deficiency 54

**DOI:** 10.1007/s10048-026-00892-5

**Published:** 2026-03-03

**Authors:** King Lam Lai, Thomas B. Smith, Reza Maroofian, Maha S. Zaki, Swetha Ramadesikan, Tamara Reynolds, Daniel C. Koboldt, Jesse M. Hunter, Jorge Vidaurre, Mihaela Atanasova, Brian D. Marsden, Wyatt W. Yue, Henry Houlden, Robert W. Taylor, William G. Newman, Raymond T. O’Keefe

**Affiliations:** 1 Division of Evolution, Infection and Genomics, School of Biological Sciences, Faculty of Biology Medicine and Health, University of Manchester, Manchester, M13 9PL United Kingdom; 2 Manchester Centre for Genomic Medicine, St Mary’s Hospital, Manchester University NHS Foundation Trust, Manchester, M13 9WL United Kingdom; 3 Department of Molecular Neuroscience, UCL Queen Square Institute of Neurology, London, WC1N 3BG United Kingdom; 4 Clinical Genetics Department, Human Genetics and Genome Research Institute, National Research Centre, Cairo, 12311 Egypt; 5 The Steve and Cindy Rasmussen Institute for Genomic Medicine, Nationwide Children’s Hospital, Columbus, OH USA; 6 Department of Pediatrics, The Ohio State University, Columbus, OH USA; 7 Division of Pediatric Neurology, Department of Pediatrics, Nationwide Children’s Hospital, The Ohio State University Wexner College of Medicine, Columbus, OH USA; 8 Centre for Medicines Discovery, Nuffield Department of Medicine, University of Oxford, Oxford, OX3 7FZ United Kingdom; 9 Faculty of Medical Sciences, Newcastle University Biosciences Institute, Framlington Place, Newcastle upon Tyne, NE2 4HH United Kingdom; 10 Mitochondrial Research Group, Clinical and Translational Research Institute, Faculty of Medical Sciences, Newcastle University, Newcastle upon Tyne, NE2 4HH United Kingdom; 11 NHS Highly Specialised Service for Rare Mitochondrial Disorders, Newcastle upon Tyne Hospitals NHS Foundation Trust, Newcastle upon Tyne, NE1 4LP United Kingdom

**Keywords:** PRORP, Mitochondria, COXPD54, Perrault syndrome, Rare disease, RNase P complex

## Abstract

Biallelic hypomorphic variants in *PRORP* cause the rare autosomal recessive disorder combined oxidative phosphorylation deficiency type 54 (COXPD54). COXPD54 encompasses a clinical spectrum of sensorineural hearing loss and ovarian insufficiency (Perrault syndrome) to leukodystrophy with developmental delay and epilepsy. Here, we report two new affected individuals with biallelic *PRORP* variants with clinical features consistent with COXPD54. One individual was homozygous for c.1505G > A p.Arg502Gln, whereas the other was compound heterozygous for c.1510C > T, p.His504Tyr and c.893C > A, p.Ser298Ter (NM_014672.4). In vitro tRNA processing assays revealed decreased mitochondrial 5′ tRNA leader cleavage by human RNase P complex with the two novel missense *PRORP* metallonuclease domain variants. These data provide further evidence that biallelic *PRORP* variants disrupt 5’ tRNA leader cleavage and are associated with a pleiotropic phenotype of COXPD54.

**Supplementary Information:**

The online version contains supplementary material available at 10.1007/s10048-026-00892-5.

## Introduction

Biallelic variants in Protein Only RNase P Catalytic Subunit (*PRORP)* are associated with combined oxidative phosphorylation deficiency 54 (COXPD54) (MIM 619737) [1, 2]. Affected individuals have clinical features, ranging from bilateral sensorineural hearing loss (SNHL) in both sexes and primary ovarian insufficiency (POI) in females (Perrault syndrome), to SNHL alone, leukodystrophy, or severe neurometabolic illness characterized by lactic acidosis and developmental delay. PRORP (MRPP3) is one of three subunits of human mitochondrial protein only RNase P (mtRNase P) complex, which catalyzes 5′ leader cleavage of polycistronic mitochondrial tRNA transcripts [3]. Here, we present two unrelated individuals with biallelic *PRORP* variants providing further independent evidence for the association of biallelic *PRORP* variants with defective mitochondrial tRNA processing, resulting in COXPD54.

## Materials and methods

All individuals (or legal guardians) provided written informed consent in accordance with local regulations. Ethical approval was granted by the NHS Ethics Committee (16/WA/0017) and University of Manchester.

Exome and Sanger sequencing confirmations with variant filtering and prioritization were performed as described and in supplemental methods [4]. The pre-tRNA processing assay has been described previously [1, 2, 5] with further details in supplemental data.

Computational models of PRORP variants p.Arg502Gln and p.His504Tyr were produced based on a cryo-electron microscopy structure of human mitochondrial RNase P (TRMT10C-SRD5C1-PRORP) in complex with mitochondrial pre-tRNA-His(5,Ser) (PDB ID: 8CBK) using PyRosetta. Structures were pre-relaxed with the *ref2015_cart* score function [6]. Single-point mutants were generated followed by local cartesian relax. Per-residue and neighbourhood energy terms were calculated (fa_atr, fa_rep, fa_sol), together with solvent-accessible surface area (ΔSASA) and ΔΔG of mutation.

## Results

Family F1 is consanguineous (distant relatives) and originates from Upper Egypt. The proband is female aged 5 years and 2 months, born to healthy parents, with three unaffected male siblings. No similar conditions were reported in the family. She presented with global developmental delay, growth retardation, and SNHL. Motor milestones were delayed; head control was achieved at 1 year, and at her most recent evaluation at 3 years, she was only able to sit with support. Cognitive function was severely impaired, with absent speech, limited environmental recognition, and a lack of sphincter control. There was no history of seizures. On physical examination, her weight was 10 kg (-4.1SD), height was 90 cm (-3.9SD), and head circumference was 47.5 cm (-2.1SD). Dysmorphic facial features were noted, including a triangular face with an open mouth, high forehead, arched eyebrows, broad nasal root and bridge, bulbous nose, long philtrum, everted lower lip, and low-set ears. Neurological examination revealed generalized hypotonia with preserved deep tendon reflexes. Investigations, including karyotyping, extended metabolic screening, urinary organic acids, thyroid profile, electromyography, nerve conduction studies, and echocardiography were unremarkable. Serum lactate was elevated at 27 mg/dL (reference 4.5–19.8), and ammonia was 68 mg/dL (reference 15–45). Auditory brainstem responses demonstrated bilateral severe-to-profound hearing loss. Brain MRI was normal, except for a thin corpus callosum and the presence of a cavum septum pellucidum. Exome sequencing identified a homozygous missense variant in *PRORP* (NM_014672.4), c.1505G > A (p.Arg502Gln). Both parents were healthy heterozygous carriers.

The proband from family F2 is a 12-year-old female of Honduran ancestry born to non-consanguineous parents who have two other clinically unaffected children. She was born at full term. She walked at 2 years and used small phrases at 2-2.5 years of age. However, there was clear regression (marked by the loss of ability to walk and talk) after she started to have multiple seizure types from around 2 years. These seizures were primarily bilateral tonic-clonic, but also tonic and hemiclonic. She has had multiple episodes of status epilepticus. Currently, she is nonverbal, non-ambulatory, has profound intellectual disability and her physical exam shows severe hypotonia and scoliosis. She has bilateral SNHL and oral dysphagia. Her brain MRI shows patchy encephalomalacia involving the right more than the left cerebral/cerebellar border zones (Figure S1-3). Seizure control has been challenging despite multiple medications. She has been seizure free for 2 months with a combination of levetiracetam and clobazam. No assessment of ovarian function has been undertaken. Trio exome sequencing of the proband revealed compound heterozygous variants in *PRORP* (NM_014672.4: c.1159 A > G, p.His504Tyr and c.893 C > A, p.Ser298Ter). No variants in other candidate genes or any of the known Perrault syndrome genes were identified. Both variants were confirmed by Sanger sequencing and segregation analysis, which reveled that her unaffected parents are heterozygous, whilst her younger brother was heterozygous for the nonsense variant and her younger sister is homozygous for the wild-type allele.

**Fig. 1:**
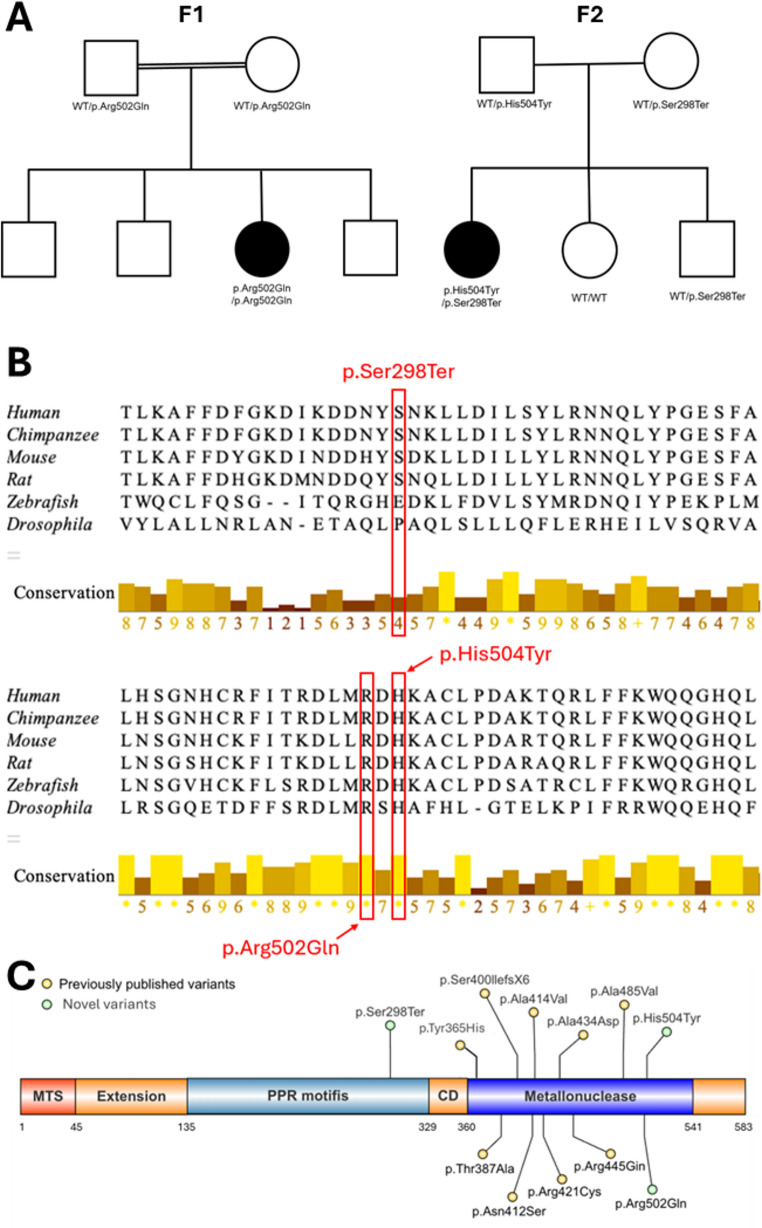
*PRORP* family pedigrees, conservation analysis of variant residues and variant locations. (**A**) Pedigrees for families F1 and F2, each with biallelic *PRORP* variants in the proband. (**B**) Position of missense variant residues highlighted in red. Symbols below the sequence alignments represent level of conservation across species, conservation scores range from 0 (least conserved) to 11 (full conservation), indicated by * (asterisk), based on AMAS method. Alignment visualised using Jalview software. (**C**) Schematic illustrating the location of all established *PRORP* variants to date (NM_014672.4). Novel variants are coloured in green, whilst previously published variants are in yellow. Figure created using IBS 2.0 [8] MTS = mitochondrial targeting sequence. PPR = pentatricopeptide repeat. CD = central domain.

The p.Ser298Ter variant is predicted to result in a null allele secondary to nonsense mediated decay. The missense variants, p.Arg502Gln and p.His504Tyr, alter highly conserved residues among PRORP orthologues (Fig. 1B) and are predicted to be deleterious by multiple in silico analyses (Table S2). Within gnomADv4.1, these variants are found at frequencies consistent with an ultra-rare autosomal recessive disorder [7].

**Fig. 2:**
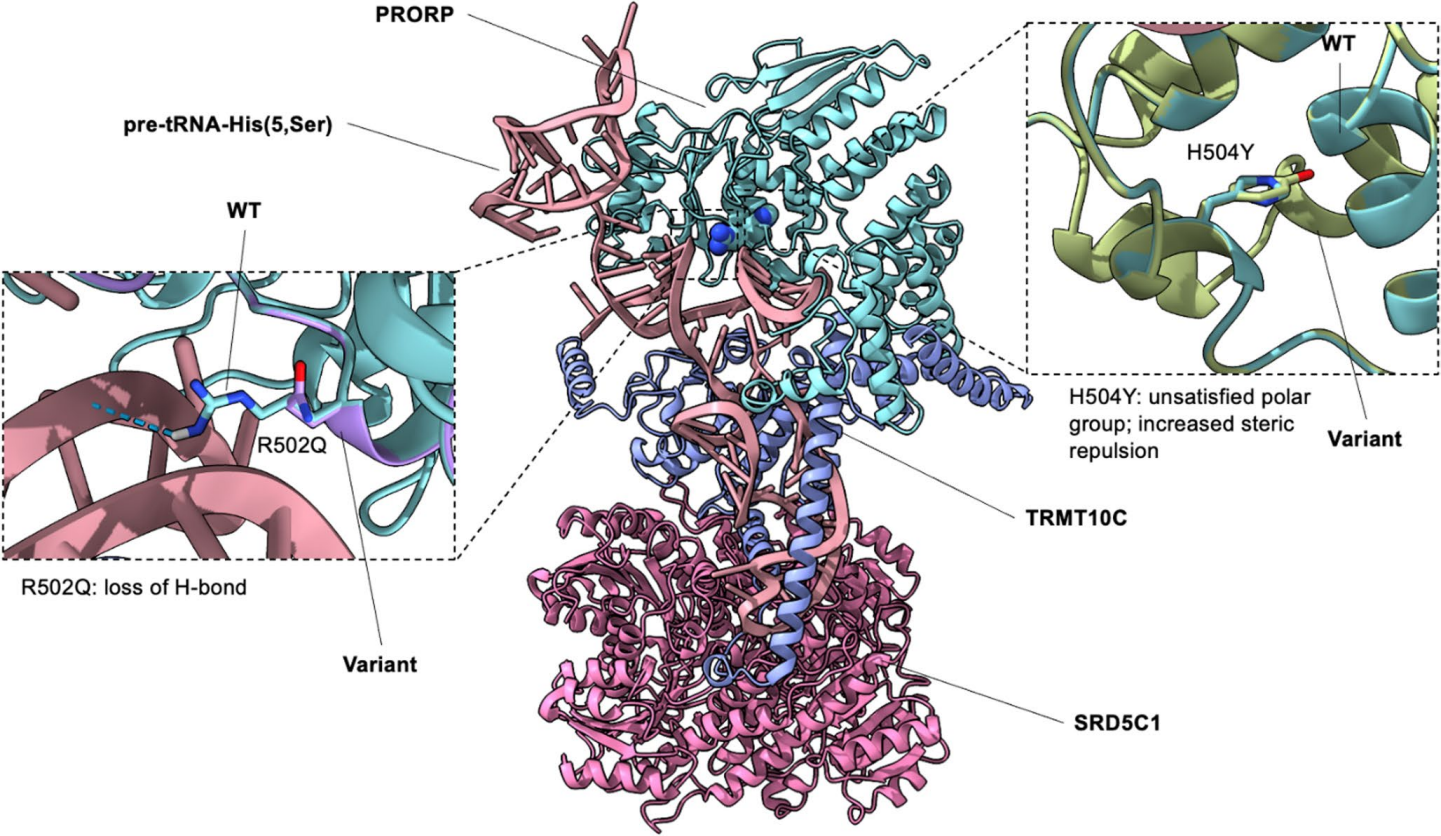
Structural analysis of PRORP variants in the context of the mtRNase P complex. PRORP variants p.Arg502Gln and p.His504Tyr modeled on the cryo-EM structure of RNase P in complex with mitochondrial pre-tRNA-His(5,Ser) (PDB 8CBK) using PyRosetta. *Inset*,* left*: p.Arg502Gln (violet side-chain) removes an RNA hydrogen bond. *Inset*,* right*: p.His504Tyr(green side-chain) fills a cavity and improves packing but increases steric repulsion and buries an unsatisfied hydroxyl. Variant side chains are shown as sticks; hydrogen bonds as dashed lines

We considered the effect of the two missense changes, by generating variant protein models of PRORP in the context of the mtRNase P complex. In the reported structure (PDB 4XGL), residue Arg502 makes direct contact with the acceptor arm and leader nucleotides of the pre-tRNA substrate, positioning the scissile phosphodiester bond in the nuclease active site [9, 10] (Fig. 2). Mispositioning of the pre-tRNA substrate may partially destabilise the optimal conformation for metallonuclease activity. p.Arg502Gln removes a hydrogen bond to pre-tRNA-His(5,Ser), weakening specific RNA contacts. In contrast, residue His504 is located close to one of the metal-binding residues (Asp503), within the metal 2 catalytic pocket [9, 10]. The p.His504Tyr substitution introduces a bulkier tyrosine that fills a cavity and improves packing but increases the steric repulsion (fa_rep term + 2.8 REU), as well as introduces one additional buried, unsatisfied polar atom from the Tyr hydroxyl. ΔSASA and ΔΔG show negligible changes in both cases.

**Fig. 3:**
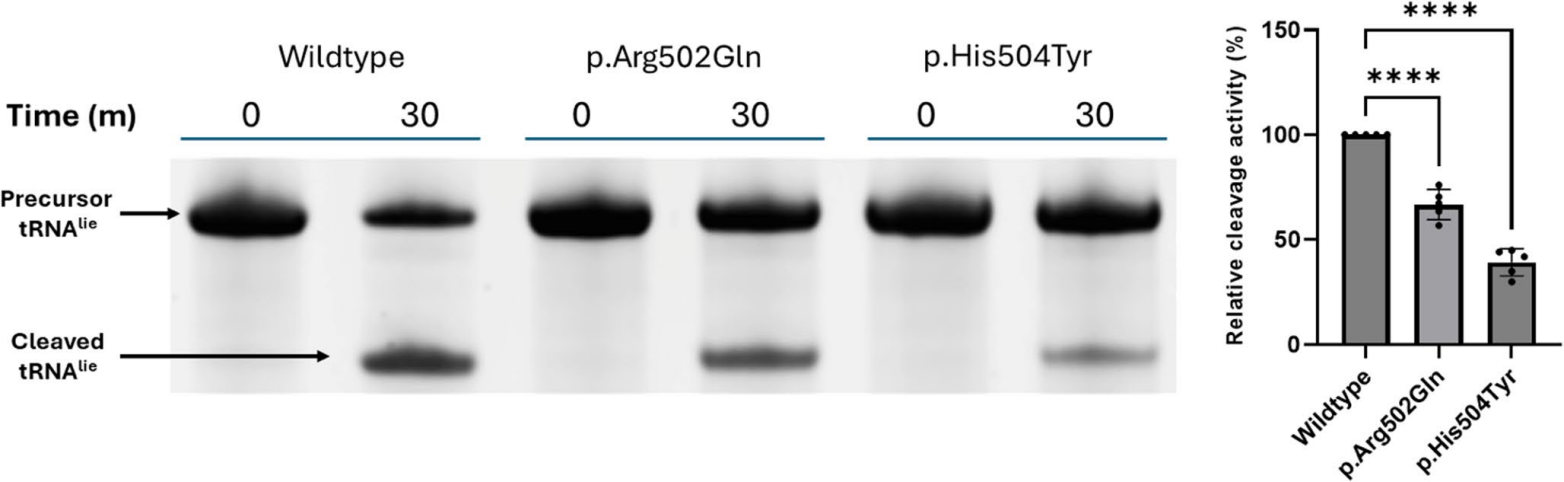
Functional assessment of PRORP variants in mtRNase P tRNA processing assays. Cleavage of the pre-tRNA^Ile^ 5′ leader sequence by mtRNase P containing wild-type (WT) or variant PRORP. The intensity of the pre-tRNA^Ile^ cleavage product was quantified, and the variants were normalized against wild-type PRORP. Error bars represent the standard error of the mean. *N* = 5, ****p < 0.0001, one-way ANOVA with Dunnett’s multiple comparisons test, comparing wild-type to variants

To determine whether the PRORP missense variants disrupted mitochondrial tRNA processing, we purified recombinant PRORP proteins carrying the variants and assessed mtRNase P complex endonucleolytic activity of pre-tRNA^Ile^ [2]. mtRNase P complexes containing the p.Arg502Gln or p.His504Tyr variants significantly diminished 5’ cleavage product levels compared to wildtype PRORP (*p* < 0.0001; Fig. 3), with relative cleavage activity decreases of 33% and 61%, respectively.

Dermal fibroblasts were not available from either affected individual to undertake OXPHOS studies or measure the levels of PRORP.

## Discussion

Biallelic *PRORP* variants are associated with the pleiotropic phenotypes of COXPD54 characterized by defective mitochondrial tRNA processing at a cellular level [1, 2]. This study reports the association and characterization of novel *PRORP* variants identified in two families. Consistent with other individuals affected with COXPD54, the missense variants are within the metallonuclease domain and patients have similar phenotypes, including SNHL, intellectual disability, and seizures with white matter changes [1, 2].

mtRNase P complexes containing the novel *PRORP* missense variants had reduced mtRNase P complex function. The defect in 5’ leader processing was greater for the p.His504Tyr variant, which was *in trans* to a null allele with a loss of function, and present in the individual with the more severe phenotype. The homozygous p.Arg502Gln variant also caused a notable processing defect, though less severe, consistent with the milder clinical presentation. These findings provide support for a correlation between mtRNase P function and phenotype severity. Notably, only one additional family harboring a nonsense allele at this locus has been reported to date [1]. In that case, a missense variant, p.Arg445Gln, was present *in trans* with a frameshift variant, p.Ser400Ilefs*6, and the affected individual also exhibited a severe childhood-onset phenotype. As the nonsense allele is predicted to generate a null allele with absence of PRORP protein, the combined reduction in total PRORP levels, i.e. the degree of PRORP loss, suggest a correlation with disease severity. COXPD54 *PRORP* variants are associated with considerable phenotypic diversity, complicating efforts to define variant-specific clinical consequences. Nonetheless, the protein structural insights provide a potential framework for categorizing these variants.

To date, COXPD54 PRORP variants can be broadly classified into three major categories based on their structural impact. The first comprises tRNA-binding/positioning variants. These variants occur within or near the tRNA-contacting residues, thereby destabilizing the optimal conformation required for 5’ cleavage activity. The tRNA-contacting residues include conserved basic residues 497–504 – encompassing the catalytic aspartates (Asp499 and Asp503), the two loops (residues 415–422 and 473–478) and a 310 helix formed by residues 446–449 [9]. The majority of COXPD54 PRORP variants lie within this region, such as p.Arg421Cys [1] and p.Arg502Gln. The second category consists of metal-binding/catalytic chemistry variants. These variants, including p.Asn412Ser in the helix region [1] and p.His504Tyr, are located close to the catalytic aspartates (Asp479/ Asp499/Asp503 and Asp409/Asp478/Asp479), where they disrupt metal-binding pocket geometry or stability [9, 10]. Interestingly, although both missense variants reported here lie near the catalytic aspartates Asp503, they influence mtRNase P function differently. Hence, positional proximity of a variant to catalytic or RNA-interacting residues does not reliably predict its structural or functional consequences, emphasizing the context-dependent impact of PRORP variants. The third category includes variants e.g. p.Thr384Ala [2], which influence the interaction between PRORP and TRMT10C, an essential component of the mtRNase P complex [9]. The interfaces between the two proteins involve PRORP residues Thr384-Lys385, which interact with the catalytic loop of TRMT10C (residue 310–320), and the PRORP pentatricopeptide repeat (PPR) domain engages with TRMT10C residues 61–106 [9]. Our work further demonstrates the value of mitochondrial tRNA processing assays to confirm the effect of *PRORP* variants when dermal fibroblasts are unavailable for functional studies supportive of the clinical phenotype of COXPD54.

In summary, these data provide further confirmation that biallelic *PRORP* missense variants reducing mitochondrial tRNA processing in vitro and are associated with variable, overlapping pleiotropic phenotypes consistent with COXPD54. These data emphasize the value of functional assays to define the effects of *PRORP* variants and expand the genotypic spectrum associated with COXPD54. 

## Supplementary Information

Below is the link to the electronic supplementary material.

Supplementary Material 1

## Data Availability

The PRORP variants were submitted to ClinVar (https://www.ncbi.nlm.nih.gov/clinvar/) (GenBank: NM_014672.4; accession numbers SCV1705405, SCV007114217 and SCV007114218.
