# Supplementary material for: Expanding the genotypic spectrum of combined oxidative phosphorylation deficiency 54
Source: Neurogenetics. 2026 Mar 3;27(1):20. doi: 10.1007/s10048-026-00892-5 (PMC12953478; doi:10.1007/s10048-026-00892-5)
Supplement: Supplementary file 1 — Supplementary Material 1 [file 10048_2026_892_MOESM1_ESM.docx]

**Supplemental Materials**

**Expanding the genotypic spectrum of combined oxidative phosphorylation deficiency 54**

King Lam Lai,^1^ Thomas B. Smith,^1,2^ Reza Maroofian^3^, Maha S. Zaki,^4^ Swetha Ramadesikan,^5^ Tamara Reynolds,^5,6^ Daniel C. Koboldt,^5,6^ Jesse M. Hunter,^5,6^ Jorge Vidaurre,^7^ Mihaela Atanasova,^8^ Brian D. Marsden,^8^ Wyatt W. Yue,^9^ Henry Houlden,^3^ Robert W. Taylor,^10,11^ William G. Newman,^1,2*^ Raymond T. O’Keefe^1,2*^

1. Division of Evolution, Infection and Genomics, School of Biological Sciences, Faculty of Biology Medicine and Health, University of Manchester, Manchester, M13 9PL, UK.

2. Manchester Centre for Genomic Medicine, St Mary’s Hospital, Manchester University NHS Foundation Trust, Manchester, M13 9WL, UK.

3. Department of Molecular Neuroscience, UCL Queen Square Institute of Neurology, London WC1N 3BG, UK.

4. Clinical Genetics Department, Human Genetics and Genome Research Institute, National Research Centre, Cairo, 12311, Egypt

5. The Steve and Cindy Rasmussen Institute for Genomic Medicine, Nationwide Children's Hospital, Columbus, OH, USA.

6. Department of Pediatrics, The Ohio State University, Columbus, OH, USA.

7. Division of Pediatric Neurology, Department of Pediatrics, Nationwide Children's Hospital, The Ohio State University Wexner College of Medicine, Columbus, Ohio, USA.

8. Centre for Medicines Discovery, Nuffield Department of Medicine, University of Oxford, Oxford, OX3 7FZ

9. Newcastle University Biosciences Institute, Faculty of Medical Sciences, Framlington Place, Newcastle upon Tyne, NE2 4HH, UK

10. Mitochondrial Research Group, Clinical and Translational Research Institute, Faculty of Medical Sciences, Newcastle University, Newcastle upon Tyne NE2 4HH, UK

11. NHS Highly Specialised Service for Rare Mitochondrial Disorders, Newcastle upon Tyne Hospitals NHS Foundation Trust, Newcastle upon Tyne NE1 4LP, UK.

Correspondence to:

[william.newman@manchester.ac.uk](mailto:william.newman@manchester.ac.uk)

[rokeefe@manchester.ac.uk](mailto:rokeefe@manchester.ac.uk)

**Supplementary methods**

**Exome sequencing**

Exome sequencing in Family F2:

The proband and her parents underwent clinical trio exome sequencing (ES) at the Steve and Cindy Rasmussen Institute for Genomic Medicine at Nationwide Children’s Hospital, Columbus, Ohio. Genomic DNA extraction was performed on peripheral blood and a custom Agena MassArray panel (Agena) was used for confirmation of sample provenance and familial relationships as described previously [1]. Libraries were prepared using NEBNextUltra II (New England Biolabs) and following target capture underwent paired-end 151bp sequencing using the NovaSeq6000 instrument (Illumina Inc., San Diego, CA, USA). Reads were mapped to the GRCh38/UCSC hg38 reference sequence and secondary data analyses were performed using Churchill [2], an in-house pipeline which implements the Genome Analysis Toolkit (GATK) best practices workflow to allow for a computationally efficient analysis of ES data. SnpEff, ANNOVAR, and custom in-house scripts were used to annotate SNPs/indels with gene, transcript, function class, damaging scores, and population allele frequencies.

Variant annotation and prioritization has been described [3]. Briefly, GATK HaplotypeCaller was used to perform multisample variant calling. Variants with a minor allele frequency [MAF] >0.001 in gnomAD and ExAC database were eliminated and all remaining missense, frameshift, nonsense, and splice site variants predicted damaging by a majority of *in silico* tools available through VarSome [3] were prioritized for further investigation. Given no family history of similarly affected individuals and presence of several unaffected siblings, we looked for rare, damaging missense or loss of function variants consistent with a Mendelian inheritance pattern – *de novo* autosomal dominant or X-linked dominant and autosomal recessive/compound heterozygous variants.

**PCR amplification and Sanger sequencing**

Following primer design targeting the regions containing the two *PRORP* variants, PCR amplification of both regions of interest was performed using the Platinum™ SuperFi II PCR Master Mix (Invitrogen, #12368010). The PCR conditions were as follows: 98 °C 30 s, 35 × (98°C 5 s, 60°C 10 s, 72°C 30 s), 72°C 5 min, 4 °C hold. PCR amplification was followed by the purification of the PCR products using the QIAquick Purification Kit (QIAGEN), which was evaluated on Agilent 4200 TapeStation per manufacturer’s protocol. Forward and reverse sequencing reactions were performed with the Big Dye v3.1 terminator mix (Thermo Fisher Scientific) and M13-tagged primers. Sequencing was performed on an Applied Biosystems 3730 instrument (Thermo Fisher Scientific). Electropherogram results using Mutation Surveyor v5.1.2.

**tRNA processing assay**

tRNA processing assays were conducted as previously described [4]. Ratio of MRPP1/MRPP2 to PRORP was optimised to 5:1. Assays were repeated independently five times, with data presented using GraphPad Prism 9 and error bars representing standard error about the mean. Statistical analysis was completed using GraphPad Prism 9, using one-way ANOVA to compare wild type to variants.

| **Variant** | **Oligonucleotide sequence – 5′ to 3′** |
| --- | --- |
| **c.1505G>A p.Arg502Gln** | CCTGATGCGGGACCAC |
| **c.1510C>T p.His504Tyr** | GGACCACAAGGCCTGT |

**Table S1:** Oligonucleotide sequences used to modify *PRORP* expression plasmid for tRNA processing assay.


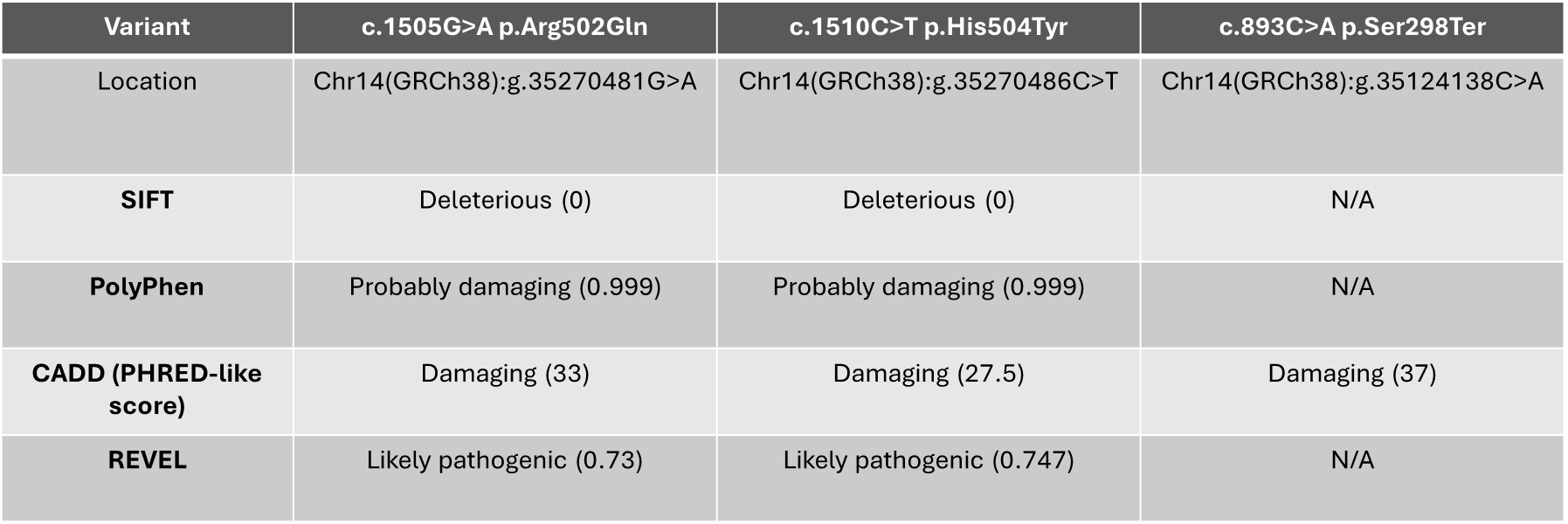


**Table S2**: *in silico* analyses of the novel *PRORP* variants.

**
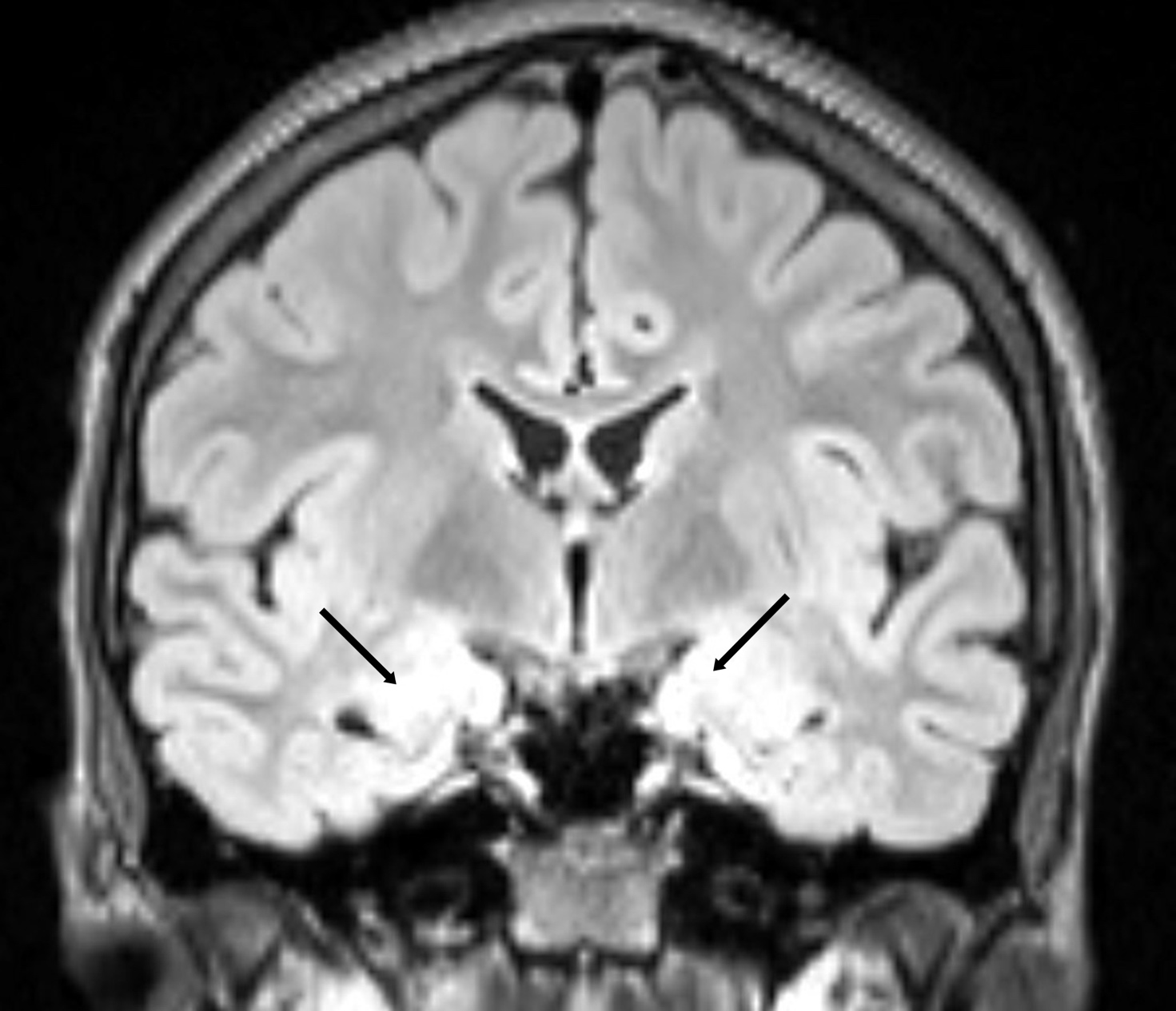
**

**Figure S1.**  T2-weighted MRI showing bilateral engorgement of the mesial temporal structures (black arrows), likely related to frequent seizures in the affected individual in F2 at age 11 years.

**
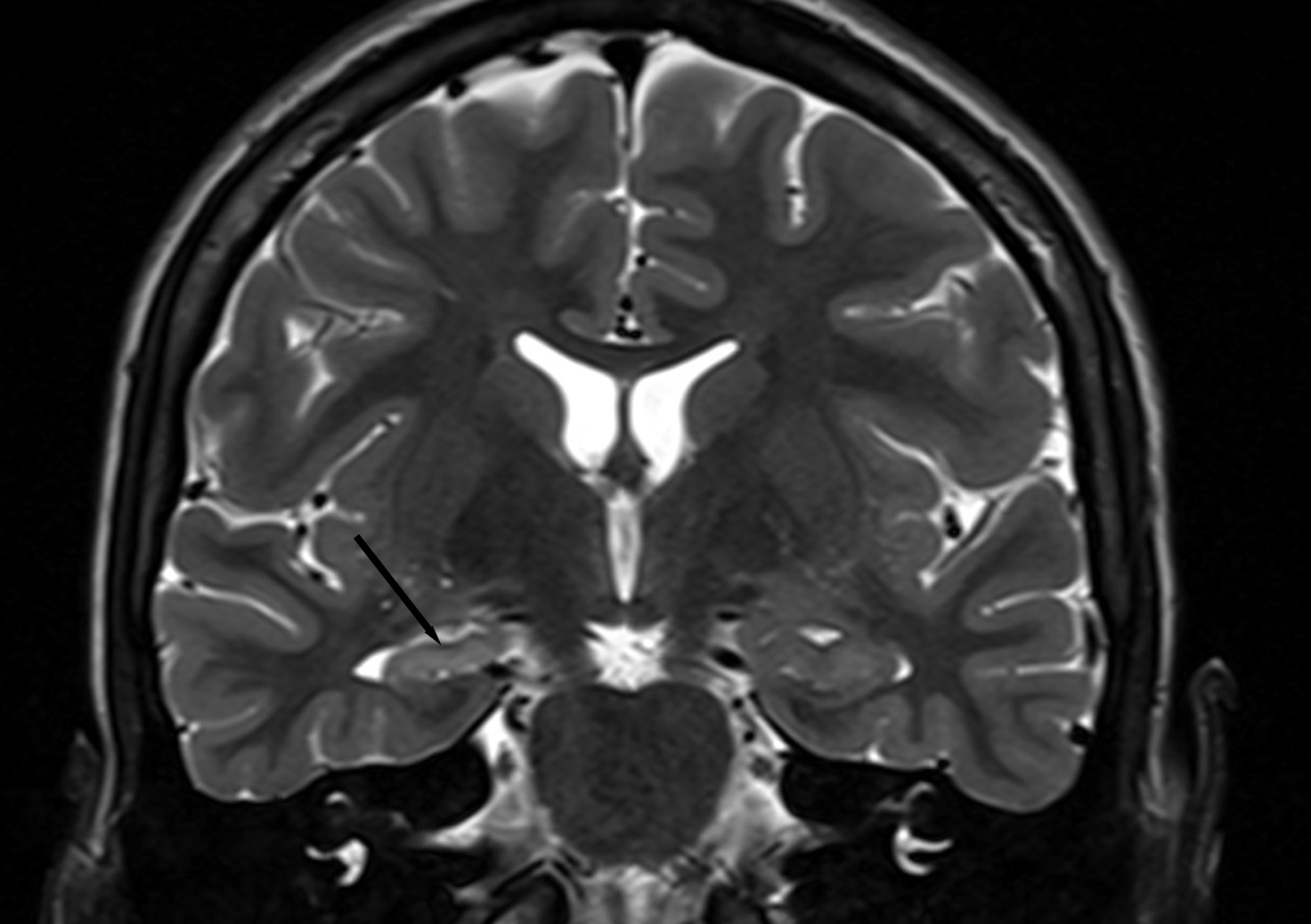
**

**Figure S2.**  Follow-up T2-weighted MRI in the affected individual in F2 at age 13 years showing atrophy and increased signal of the right mesial temporal structures, consistent with mesial temporal sclerosis (black arrow). This is likely a result of frequent seizures.


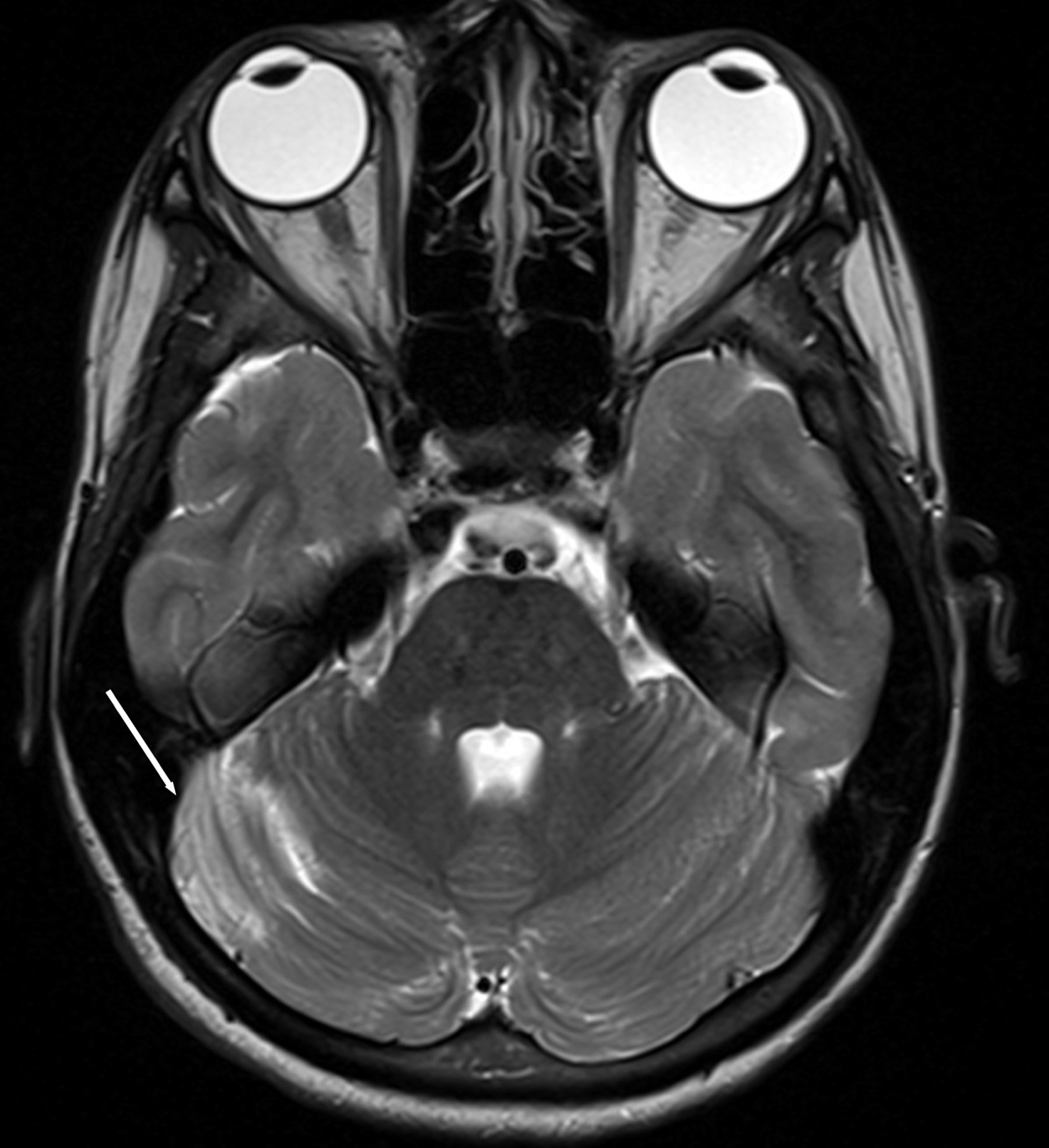


**Figure S3.** Brain MRI demonstrating atrophy of the right cerebellum, probably due to nonspecific gliotic changes (white arrow) in the affected individual in F2 at age 13 years.

**References**

1. Miller CR, Lee K, Pfau RB, Reshmi SC, Corsmeier DJ, Hashimoto S, Dave-Wala A, Jayaraman V, Koboldt D, Matthews T, Mouhlas D, Stein M, McKinney A, Grossman T, Kelly BJ, White P, Magrini V, Wilson RK, Mardis ER, Cottrell CE. Disease-associated mosaic variation in clinical exome sequencing: a two-year pediatric tertiary care experience. Cold Spring Harb Mol Case Stud. 2020 Jun 12;6(3):a005231. doi: 10.1101/mcs.a005231.
2. Kelly, Benjamin J et al. “Churchill: an ultra-fast, deterministic, highly scalable and balanced parallelization strategy for the discovery of human genetic variation in clinical and population-scale genomics.” *Genome biology* vol. 16,1 6. 20 Jan. 2015, doi:10.1186/s13059-014-0577-x
3. Koboldt DC, Mihalic Mosher T, Kelly BJ, Sites E, Bartholomew D, Hickey SE, McBride K, Wilson RK, White P. A de novo nonsense mutation in ASXL3 shared by siblings with Bainbridge-Ropers syndrome. Cold Spring Harb Mol Case Stud. 2018 Jun 1;4(3):a002410. doi: 10.1101/mcs.a002410.
4. Smith TB, Rea A, Thomas HB, Thompson K, Oláhová M, Maroofian R, et al. Novel homozygous variants in PRORP expand the genotypic spectrum of combined oxidative phosphorylation deficiency 54. Eur J Hum Genet. 2023 Oct;31(10):1190-1194.
